# How Much Weight Loss is Effective on Nonalcoholic Fatty Liver Disease?

**DOI:** 10.5812/hepatmon.15227

**Published:** 2013-12-07

**Authors:** Alireza Ghaemi, Fourugh Azam Taleban, Azita Hekmatdoost, Alireza Rafiei, Vahid Hosseini, Zohreh Amiri, Reza Homayounfar, Hafez Fakheri

**Affiliations:** 1 Department of Clinical Nutrition and Dietetics, National Nutrition and Food Technology Research Institute, Faculty of Nutrition Sciences and Food Technology, Shahid Beheshti University of Medical Sciences, Tehran, IR Iran; 2 Molecular and Cell Biology Center, Faculty of Medicine, Mazandaran University of Medical Sciences, Sari, IR Iran; 3 Inflammatory Diseases of Upper Gastrointestinal Tract Research Center, Mazandaran University of Medical Sciences, Sari, IR Iran; 4 Department of Basic Sciences, National Nutrition and Food Technology Research Institute, Faculty of Nutrition Sciences and Food Technology, Shahid Beheshti University of Medical Sciences, Tehran, IR Iran

**Keywords:** Nonalcoholic Fatty Liver Disease, Diet, Weight Loss

## Abstract

**Background:**

Nonalcoholic fatty liver disease (NAFLD) is the most common liver disease worldwide with no specific treatment. Weight loss is the most effective therapeutic strategy in its management; however, there is no consensus on its specifics. Thus, this study was conducted to evaluate the effects of weight loss on liver enzymes, markers of inflammation, oxidative stress and CK18-M30 (cytokeratin 18) as a biomarker of hepatocellular apoptosis.

**Objectives:**

To study the effect of weight reduction diet as an exclusive treatment for NAFLD.

**Patients and Methods:**

Forty four patients with NAFLD received a diet including a 500 to 1000 kcal per day intake reduction as30% fat, 15% protein, and 55% carbohydrate for six months. Anthropometric parameters, alanine aminotransferase (ALT), aspartate aminotransferase (AST), gamma glutamyl transferase (GGT), lipid profile, malondialdehyde (MDA), TNF-α, IL-6, CK18-M30 were measured at baseline and at the end of the study. At the end of follow up, patients were classified as adherent or nonadherent to treatment according to a weight loss of ≥ 5%, or < 5% of initial body weight, respectively.

**Results:**

Twenty five patients were classified as adherent group and nineteen as nonadherent group (9.7% vs. 1.9% total body weight loss after 6 months, respectively). After 6 months, changes in adherent and nonadherent groups were as follows: reduction in body weight from 93.7 ± 15.8 kg to 84.2 ± 13.4 kg vs. 94 ± 16.6 kg to 92.2 ± 16.2 kg (P < 0.05), BMI from 32.7 ± 3.9 to 29.5 ± 3.2 vs.31.8 ± 5.4 to 31.1 ± 5.3 (P < 0.001), and waist circumference from 105.1 ± 12.6 cm to 97.4 ± 9.8 cm vs.106.8 ± 14.2 cm to 103.7 ± 14 cm (P < 0.001), respectively. Diastolic blood pressure was significantly decreased in adherent group (from 80.2 ± 5.1 mmHg to 76.9 ± 5 mmHg; P < 0.001). Also, total cholesterol, LDL, triglyceride, ALT, AST, GGT and CK18-M30 levels were significantly decreased in the adherent group compared to nonadherent group (P < 0.05).

**Conclusions:**

This intervention offers a practical approach for treatment of patients with NAFLD with diet therapy.

## 1. Background

Nonalcoholic fatty liver disease (NAFLD) is characterized by accumulation of fat in liver when it exceeds 5-10% of its weight (1-3). It is a spectrum of liver disease ranging from simple fatty infiltration of liver parenchyma (steatosis) to an inflammatory progression to nonalcoholic steatohepatitis (NASH) and ultimately cirrhosis (3-6). Estimates suggest that about 34% to 46% of the general adult population and 70-80% of obese individuals in western countries have some degrees of NAFLD (7, 8). The prevalence of NAFLD in an Iranian adult general population has been reported as high as 21.5% to 31.5% (9-11). If the diagnosis is not established in early stages of the disease, it would be converted to a major health burden. It is also associated with a large amount of health care cost (3, 12-14). The efficacy and safety profile of pharmacotherapy in the treatment of NAFLD has yet been remained uncertain (15-17). There is no effective specific therapy for NAFLD. The most acceptable strategy in the management of these patients is the use of diet to decrease body weight, but there are limited data in details of diet modification such as how, how much and how rapidly to lose weight (5, 16-20). Furthermore, precise hepatic and extra hepatic benefits of weight loss are not well defined (5, 21, 22).

## 2. Objectives

Since there is no consensus on the specifics of weight loss for management of NAFLD, this single-arm trial study was conducted to evaluate the effects of weight loss on NAFLD characteristics and some related conditions while addressing some of its mechanism of action.

## 3. Patients and Methods

We are trying to detect a mean difference of Δ = 13 IU/L for a variable ALT with a standard deviation of 21 IU/L (19). Assuming a type one error of 5% and a type two error of 20%, the sample size was then calculated to be 43 subjects. Forty four patients with NAFLD were recruited from two GI clinics in Sari, Mazandaran, Iran between October 2012 to July 2013. We used convenience sampling technique. The diagnosis of NAFLD was established by the presence of steatosis of the liver on ultrasound associated with a persistent increase in alanine aminotransferase (ALT), and aspartate aminotransferase (AST) of at least 1.5 times the limit of normal and body mass Index (BMI) between 25 and 40. Also, they were all tested for HBS-Ag, HBc-Ab, HCV antibody, antinuclear antibody (ANA), antimitochondrial antibody (AMA), anti-smooth muscle antibody (ASMA), anti-liver-kidney-microsomal antibody (anti LKM), Ceruloplasmin and ferritin to rule out the presence of other liver diseases. Other exclusion criteria were: 1) alcohol consumption; 2) patient receiving hepatotoxic and insulin-sensitizing medication; 3) cigarette smoking; 4) weight reduction surgery within the past year, or used weight loss medication or program in the previous 3 months. All patients signed a written informed consent, and the study protocol was approved by the Ethics Committee of National Nutrition and Food technology Research Institute, Shahid Beheshti University of Medical Sciences, Tehran, Iran with the ethic number of 043434.

### 3.1. Dietary Intervention and Anthropometric Data

All patients received an individual nutritional instruction. The diet included a reduction of 500 to 1000 kcal/day regarding the Adjusted Ideal Body Weight containing 30% fat, 15% protein, and 55% carbohydrate. Anthropometric measurement (height, weight, waist/hip diameter) and blood pressure were collected by a trained field worker while patient wore light clothing with no shoes. Height was measured by Seca stadiometer, readout accuracy: 0.5 cm. weight was measured by Seca scale, readout accuracy: 100 g. Body mass index (BMI) was calculated as weight divided by height squared (kg/m²). Waist circumference was measured with the subject standing and wearing only under wear, at the level midway between the lower rib margin and iliac crest, and hip circumference at the widest portion of buttock. Waist to hip ratio (WHR) was calculated by dividing waist circumference by hip circumference. Nutritional intake was assessed using three 24-hourrecall (including 2 work days, and 1 weakened). Physical activity was evaluated using the Metabolic Equivalent of Task (MET) questionnaire at first, and the end of months 3, and 6. The questionnaire had been previously modified and validated among an Iranian young people (23, 24).

### 3.2. Laboratory Tests

Blood samples were obtained from all patients after an overnight fasting (12-14 hours) at the beginning of the study, and at the end of months 3 and 6. Serum fasting glucose, alanine aminotransferase (ALT), aspartate aminotransferase (AST), gamma glutamyl transferase (GGT), total cholesterol, low density lipoprotein (LDL), high density lipoprotein (HDL), triglycerides were measured using Hitachi autoanalyzer 911 (Japan) with ParsAzmun reagents kits (Tehran, Iran). The homeostasis model assessment for insulin resistance (HOMA-IR) value was calculated as fasting glucose (mg/dL) multiplied by fasting insulin (μU /mL)/405, and the quantitative insulin-sensitivity check index (QUICKI) was calculated as 1/[ log fasting insulin(μU/mL) + log fasting glucose (mg/dL) ] (25).Serum was frozen at -80ºCto measure Insulin, malondialdehyde (MDA), tumor necrosis factor-α (TNF-α), interleukin-6 (IL-6), and cytokeratin 18 –M30 (CK18-M30). After a single thawing, assays were performed using enzyme-link immunosorbent assays. Insulin, MDA, TNF-α, IL-6 and CK18-M30 were measured using Elisa kits DIAPLUS (North York, Canada), GLORY (Del Rio, TX, USA), Orgenium (Vantaa Finland), Orgenium (Vantaa Finland), and PEVIVA (Bromma, Sweden) respectively.

### 3.3. Follow Up

The intervention was weight loss within 6 months. The participants were visited monthly by an expert nutritionist and were also visited by a gastroenterologist at baseline and after 3 and 6 months. During the follow up visits, each subject received individual nutrition counseling to achieve dietary goals (3, 17, 18). Anthropometric, dietary intake, physical activity and all blood tests were assessed at the beginning and the end of the study; furthermore, anthropometric, dietary intake, physical activity and liver enzymes were evaluated at the end of the third month (26).

### 3.4. Statistical Analysis

To examine the normal distribution of quantitative variables, Shapiro–Wilk test was used. Baseline parameters were compared between the groups using Mann-Whitney U test and for comparing the variables before and after therapy, Wilcoxon signed ranks test was used for variables that were not normally distributed. Student’s t-tests were used for normal distributed parametric quantitative data. In addition, repeated measure test was also used to compare mean values within the groups. Moreover, to analyze the three days food records, Nutritionist IV software was used. SPSS software package v.20 (Chicago, IL) was used for all the analyses.

All p -values were two-tailed, and a P value < 0.05 was considered significant. 

## 4. Results

### 4.1. Characteristics of the Participants

**Figure 1.**
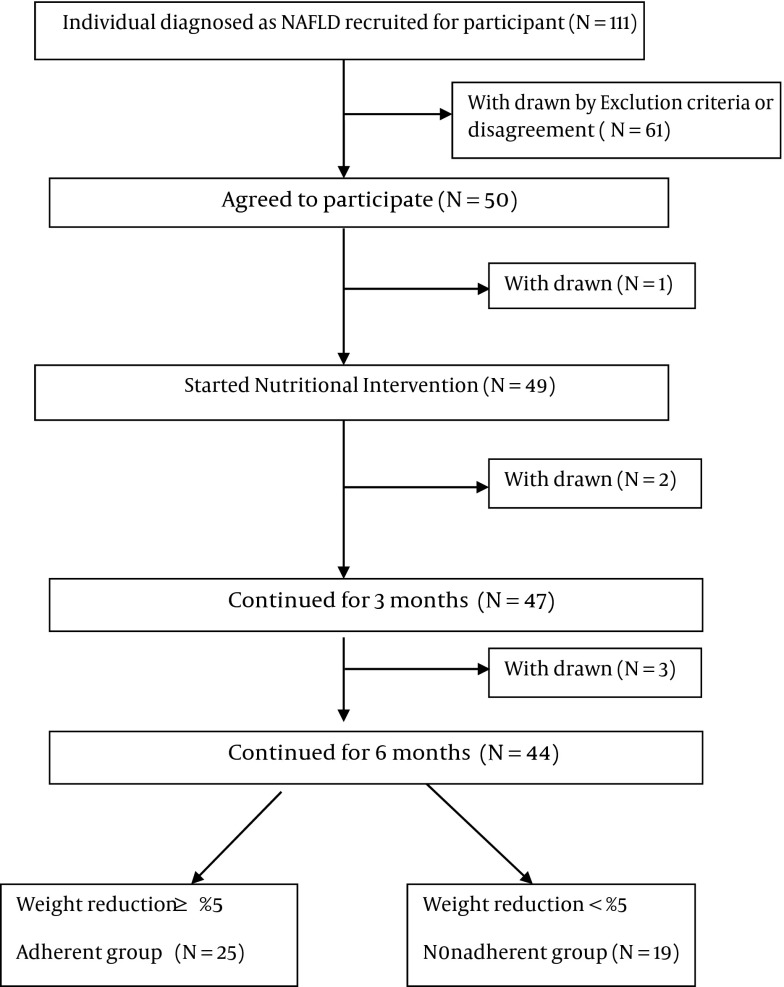
The Recruitment Process of the Study

From October 2012 to July 2013, 50 patients were initially invited to participate in this 6-month intervention program, but six were lost to follow up (Figure 1). 

A total of 44 patients, 28 men and 16 women, completed the 6-month intervention. The mean age was 36.9 ± 8.8 years. At the end of the 6th month of intervention, 25 participants were classified as adherent, and 19 as nonadherent according to weight loss (≥ 5% or < 5% of initial bodyweight) (2, 19). 

The baseline characteristics of adherent and nonadherent groups are shown in Table 1. Only GGT levels significantly differed between the two groups at the beginning of the study (P = 0.006). The variation of GGT concentration from the beginning of the study to the end was significantly different between the two groups (-12.8 ± 20.4) in adherent group vs. 2.7 ± 11 in nonadherent group, (P < 0.002). 

**Table 1.** Baseline Demographic, Anthropometric and Biochemical Characteristics of Patients ^a^

Characteristic	Adherent (n = 25)	Non-adherent (n = 19)	P value
**Male/Female, No.**	15/10	13/6	0.753
**Age, y**	38 ± 9.9	35.4 ± 7.2	0.328
**Weight, kg**	93.7 ± 15.8	94 ± 16.6	0.950
**BMI , kg/m²** ^**b**^	32.7 ± 3.9	31.8 ± 5.4	0.556
**Waist circumference, cm**	105.1 ± 12.6	106.8 ± 14.2	0.676
**WHR ** ^**b**^	0.92 ± 0.08	0.96 ± 0.08	0.181
**SBP , mmHg** ^**b**^	120.4 ± 8.9	118.9 ± 5.7	0.537
**DBP , mmHg** ^**b**^	80.2 ± 5.1	79.7 ± 4.2	0.750
**ALT , IU/L** ^**b**^	87.1 ± 40.8	69.1 ± 32.4	0.122
**AST , IU/L** ^**b**^	51.9 ± 25.2	42.7 ± 12	0.121
**GGT , IU/L** ^**b**^	56.1 ± 36.8	32.9 ± 12.9	0.006
**FBG , mg/dL** ^**b**^	98 ± 19.7	109.3 ± 45	0.329
**Fasting insulin, IU/mL**	12.9 ± 21.1	18.6 ± 28.8	0.345
**HOMA-IR ** ^**b**^	2.9 ± 4.3	4.9 ± 6.4	0.124
**QUICKI ** ^**b**^	0.36± 0.05	0.34 ± 0.06	0.336
**TCHO , mg/dL** ^**b**^	207.8 ± 42.1	206.7 ± 45.3	0.932
**HDL-C , mg/dL** ^**b**^	46.4 ± 9.9	44.8 ± 14.9	0.668
**LDL-C , mg/dL** ^**b**^	120.4 ± 34.9	111.2 ± 34.7	0.389
**TG ** ^**b**^ **, mg/dL**	195.7 ± 94.5	203.8 ± 124.9	0.807
**MDA ** ^**b**^ **, nmol/mL ** ^**c**^	15.3 (12.9–27.7)	14.5 (13–31.6)	0.804
**TNF-α ** ^**b**^ **, pg/mL ** ^**c**^	39.8 (16.2–48.2)	38.4 (19.5–61.3)	0.991
**IL-6 ** ^**b**^ **, pg/mL ** ^**c**^	0.33 (0.16–31.9)	0.19 (0.16–6)	0.943
**CK18- M30 ** ^**b**^ **, U/L ** ^**c**^	460.3 (380.6–649.4 )	404.9 (258.5–543.7)	0.181

^a^Values are expressed as mean ± SD.

^b^ Abbreviations: ALT, Alanine aminotransferase; AST, Aspartate aminotransferase; BMI, Body mass index; CK18- M30, Cytokeratin 18- M30; DBP, Diastolic blood pressure; FBG, Fasting blood glucose; GGT, γ-glutamyltransferase; HDL-C, High- density lipoprotein cholesterol; HOMA-IR, Homeostasis model assessment of insulin resistance; IL-6, Interleukin-6; LDL-C, Low- density lipoprotein cholesterol; MDA, Malondialdehyde; QUICKI, quantitative insulin-sensitivity check index; SBP, Systolic blood pressure; TCHO, Total cholesterol; TG, Triglyceride; TNF-α, Tumor necrosis factor- α; WHR, Waist to hip ratio.

^c^ Median (Interquartile range), Mann-Whitney test.

### 4.2. Anthropometric and Clinical Parameters 

The anthropometrics and clinical parameters showed significant improvement in weight loss, BMI, waist circumference and WHR in the two groups after 3 and 6 months of intervention. But diastolic blood pressure (DBP) decreased significantly only in adherent group (P < 0.001) (Table 2). 

**Table 2.** Anthropometric, Clinical Parameters and Liver Enzyme Level for Adherent and Nonadherent Patients at Baseline and After 3 and 6 Months of Nutritional Intervention ^a^

Characteristic	Adherent (n = 25)			Non adherent (n = 19)		
	Baseline	3 months	6 months	baseline	3 months	6 months
**Weight, kg**	93.7 ± 15.8	86.6 ± 13.5 ^c^	84.2 ± 13.4 ^c, d^	94 ± 16.6	92.1 ± 16.5 ^c^	92.2 ± 16.2 ^e^
**BMI , kg/m²** ^**b**^	32.7 ± 3.9	30.4 ± 3.3 ^c^	29.5 ± 3.2^ c, d^	31.8 ± 5.4	31.2 ± 5.4 ^c^	31.1 ± 5.3^c^
**Waist circumference, cm**	105.1 ± 12.6	99.5 ± 10.4 ^c^	97.4 ± 9.8^ c, d^	106.8 ± 14.2	104.3 ± 14 ^c^	103.7 ± 14 ^c^
**WHR ** ^**b**^	0.92 ± 0.08	0.90 ± 0.06 ^e^	0.90 ± 0.06	0.96 ± 0.08	0.95 ± 0.08	0.94 ± 0.08^e^
**SBP , mmHg** ^**b**^	120.4 ± 8.9	120.6 ± 5.4	118.1 ± 6.3	118.9 ± 5.7	117.9 ± 6.3	117.8 ± 7.1
**DBP , mmHg** ^**b**^	80.2 ± 5.1	81.2 ± 2.6	76.9 ± 5 ^c, d^	79.7 ± 4.2	78.4 ± 3.7	77.5 ± 4.5
**ALT , IU/L** ^**b**^	87.1 ± 40.8	45.9 ± 23.6 ^c^	45.6 ± 19.8 ^c^	69.1 ± 32.4	48.9 ± 21.7 ^c^	57.8 ± 33.1
**AST , IU/L** ^**b**^	51.9 ± 25.2	31.5 ± 11.9 ^c^	31.3 ± 11.4 ^c^	42.7 ± 12	34.3 ± 12.2 ^e^	39.5 ± 19.4
**GGT , IU/L** ^**b**^	56.1 ± 36.8	43.4 ± 33.9 ^c^	43.2 ± 31 ^c^	32.9 ± 12.9	31.8 ± 11.9	35.7 ± 11.9

^a^Values are expressed as mean ± SD.

^b^Abbreviations: ALT, Alanine aminotransferase; AST, Aspartate aminotransferase; BMI, Body mass index; DBP, Diastolic blood pressure; GGT, γ-glutamyltransferase; SBP, Systolic blood pressure; WHR, Waist to hip ratio.

^c^P< 0.001 Comparison with baseline within group.

^d^P< 0.001 Comparison with month 3 within group.

^e^P< 0.05 Comparison with baseline within group.

At the end of 6th month, the participants in adherent group lost an average of 9.7% (9.5 ± 8.6 Kg) of baseline weight vs. 1.9% (1.9 ± 2.8 Kg) in nonadherent group (P < 0.05).

### 4.3. Glucose, Insulin and Lipid Profile

After 6 months intervention neither adherent group nor nonadherent showed any significant change in Fasting blood glucose (FBG), Fasting serum insulin, HOMA-IR, QUICKI, but total cholesterol (P = 0.004), LDL (P = 0.007) and Triglyceride levels (P = 0.035) in adherent group were significantly improved when compared to the nonadherent group (Table 3). 

**Table 3.** Comparison of Glucose, Insulin, HOMA-IR, QUICKI, Lipid Profile, MDA, CK18-M30 and Markers of Inflammation Between Adherent and Nonadherent Patients at Baseline and After 6 Months Nutritional Intervention ^a^

Characteristic	Adherent (n = 25)		P value ^c^	Non-adherent (n = 19)		P value ^c^	P value ^e^
	Baseline	6 months		baseline	6 months		
**FBG ^b^, mg/dL**	98 ± 19.7	90.8 ± 13.8	0.056	109.3 ± 45	109.9 ± 62.1	0.912	0.143
**Fasting Insulin, μIU/mL**	12.9 ± 21.1	12.9 ± 13.1	0.991	18.6 ± 28.8	11.6 ± 10.9	0.322	0.705
**HOMA-IR ** ^**b**^	2.9 ± 4.3	2.9 ± 2.9	0.965	4.9 ± 6.4	3.1 ± 3.1	0.226	0.8
**QUICKI ** ^**b**^	0.36 ± 0.05	0.35 ± 0.06	0.587	0.34 ± 0.06	o.35 ± 0.05	0.742	0.792
**TCHO , mg/dL** ^**b**^	207.8 ± 42.1	181.3 ± 38.3	0.004	206.7 ± 45.3	191.7 ± 39	0.056	0.381
**HDL-C , mg/dL** ^**b**^	46.4 ± 9.9	42.7 ± 7.4	0.057	44.8 ± 14.9	41.8 ± 10.9	0.295	0.730
**LDL-C , mg/dL** ^**b**^	120.4 ± 34.9	99.9 ± 26.5	0.007	111.2 ± 34.7	102.3 ± 26.8	0.113	0.770
**TG , mg/dL** ^**b**^	195.7 ± 94.5	159.8 ± 84.2	0.035	203.8 ± 124.9	221.7 ± 144.8	0.22	0.082
**MDA , nmol/mL ** ^**b**^ ^**d**^	15.3 (12.9-27.7)	17.4 (13.9-40.2)	0.313	14.5 (13– 31.6)	15.2 (13.5-31.5)	0.748	0.915
**CK-18 M30 , U/L ** ^**b**^ ^**d**^	460.3 (380.6-649.4 )	370.7 (298.4-468.3)	0.003	404.9 (258.5 – 543.7)	469 (276.9-704.9)	0.295	0.213
**TNF-α , pg/mL ** ^**b**^ ^**d**^	39.8 (16.2– 48.2)	39.9 (10.5-49)	0.737	38.4 (19.5 – 61.3)	26.3 (9.4-57.8)	0.184	0.594
**IL-6 , pg/mL ** ^**b**^ ^**d**^	0.33 (0.16– 31.9)	0.45 (0.18-48.4)	0.323	0.19 (0.16 – 6)	0.34 (0.16-85.3)	0.001	0.831

^a^Values are expressed as mean ± SD.

^b^Abbreviations: CK-18 M30, Cytokeratin 18- M30; FBG, Fasting blood glucose; HDL-C, High-density lipoprotein cholesterol; HOMA-IR, Homeostasis model assessment of insulin resistance; IL-6, Interleukin-6; LDL-C, Low-density lipoprotein cholesterol; MDA, Malondialdehyde; QUICKI, Quantitative insulin-sensitivity check index; TCHO, Total cholesterol; TG, Triglyceride; TNF-α, Tumor necrosis factor- α.

^c^P value within group.

^d^ Median (Interquartile range),Wilcoxon signed rank test within group and Mann-Whitney test between the groups at 6th month.

^e^P value between group at month 6.

### 4.4. Liver Enzyme

In adherent group ALT, AST and GGT were significantly decreased after 3 and 6 months of intervention when compared to the baseline levels (P < 0.001). But in nonadherent group, ALT (P = 0.003) and AST (P = 0.016) were significantly decreased only after the third month in comparison to baseline levels (Table 2). 

### 4.5. MDA, CK18-M30 and Markers of Inflammation

CK18-M30 as a biomarker of hepatocellular apoptosis was significantly decreased from baseline level only in adherent group (P = 0.003).

In both groups MDA increased and TNF-α decreased slightly (but not significantly). IL -6 tended to increase with intervention, but was significantly increased only in the nonadherent group (P = 0.001) (Table 3). 

### 4.6. Dietary Intakes

There was no difference in dietary intakes between the two groups at baseline. Analysis of food intakes by patients at baseline, 3rd, and 6th month reflected the amount of weight loss. All subjects who achieved ≥ 5 % or,<5% weight loss presented a decrease in calorie intake (kcal) (P < 0.001), protein (g) (P < 0.001) , carbohydrate(g) (P < 0.001), total sugar (g) (P < 0.001), sucrose (g) (P < 0.05), total fat (g) (P < 0.001), saturated fat (g) (P < 0.001)mono-unsaturated fatty acids (MUFA) (g), and poly-unsaturated fatty acids (PUFA) (g). Selenium and fiber intake were reduced significantly in the both groups, but all were in the normal range during the study. 

β- Carotene intake increased significantly in the both groups (P < 0.001). The intake of vitamin A-RAE was decreased and alphacarotene increased none significantly in the both groups. Vitamin C intake in nonadherent group was significantly decreased; however, it was always in the normal Dietary Reference Intake (DRI) range. Vitamin E intake was reduced significantly in the adherent group. Fructose intake was significantly decreased in adherent group at months 3 and 6, but in nonadherent group, it was significantly decreased at month 3, but not after the 6th month.

No difference in the patients’ physical activity was seen at baseline and after 3 and 6 months of dietary intervention in the both groups (Table 4). 

**Table 4.** Physical Activity and Daily Food Intake of Patients at Baseline, 3 and 6 Months After the Nutritional Intervention ^a^

Characteristic	Adherent (n = 25)			Non-adherent (n = 19)		
	baseline	3 months	6 months	baseline	3 months	6 months
**Activity, MET**	1.3 ± 0.1	1.3 ± 0.1	1.3 ± 0.1	1.2 ± 0.2	1.2 ± 0.2	1.2 ± 0.2
**Energy, Kcal/d**	2888.7 ± 984.9	20271 ± 641.2 ^c^	2015.1 ± 676.2 ^c^	3021.6 ± 953.9	2380.6 ± 747.9 ^c^	2301.9 ± 692.8 ^c^
**Protein, g/d**	123.3 ± 47	95.4 ± 33.9 ^c^	92.8 ± 33.3 ^c^	134.6 ± 51.3	105.9 ± 37.6 ^c^	104.8 ± 35.7 ^c^
**Carbohydrates, g/d**	450 ± 147.9	303 ± 99.7 ^c^	305 ± 104.9 ^c^	445.9 ± 150.3	346.3 ± 119.9 ^c^	338.9 ± 88.8 ^c^
**Sugar total, g/d**	171.8 ± 63.6	127 ± 40.7 ^c^	121.4 ± 38.9 ^c^	176.8 ± 66.9	128.5 ± 50.3 ^c^	132.1 ± 30.3 ^c^
**Fructose, g/d**	29 ± 16.4	21.3 ± 10 ^d^	18.7 ± 8.1 ^c^	28.1 ± 16.6	21 ± 11.6 ^d^	22.6 ± 7.3
**Sucrose, g/d**	34.4 ± 20.7	27.1 ± 17.2	21 ± 11.9 ^c^	29.6 ± 16.4	16.9 ± 11.3 ^c^	19.5 ± 10.2 ^d^
**Fiber–total, g/d**	47 ± 15.4	34.6 ± 10.9 ^c^	34.6 ± 13.9 ^c^	46.4 ± 22.6	38.5 ± 21	35.6 ± 12.3 ^d^
**Total Fat , g/d** ^**b**^ **, g/d**	74.3 ± 28.4	54.4 ± 17.7 ^c^	53.7 ± 19.9 ^c^	84.1 ± 31.8	69.8 ± 27.4 ^d^	65.3 ± 30.5 ^c^
**Sat Fat, g/d**	23 ± 9.2	18 ± 5.9 ^c^	17.8 ± 6.7 ^c^	26.4 ± 9.3	22.4 ± 7.6 ^d^	20.2 ± 8.9 ^c^
**MUFA , g/d** ^**b**^ **, g/d**	27 ± 11.2	19.6 ± 7.4 ^c^	18.9 ± 7.6 ^c^	30.1 ± 12	25 ± 10.7 ^d^	23.9 ± 11.4 ^d^
**PUFA , g/d** ^**b**^ **, g/d**	15.2 ± 6.6	10.7 ± 4.4 ^c^	10.8 ± 4.7 ^c^	18.3 ± 8.9	14 ± 0.7 ^d^	13.8 ± 7 ^d^
**Cholesterol, mg/d**	345.1 ± 231.6	245.2 ± 139.1 ^d^	254.7 ± 140.7	469 ± 369.9	318 ± 218	319.6 ± 202.1
**Vita A, RAE/d**	782.7 ± 1782.7	229.4 ± 149.5	416.3 ± 542	663.8 ± 1115.2	576 ± 1186.5	566.8 ±1035.4
**Beta carotene, μg/d**	764.9 ± 789.5	1096.9 ± 1455.6	1682.2 ± 1535.8 ^c^	579.5 ± 610.9	655.9 ± 456.8	1350.9 ± 882.8 ^c^
**Alphacarotene, μg/d**	172.4 ± 305.3	270.3 ± 553.9	219.1 ± 538.8	92.8 ± 100.9	91.6 ± 114.9	128.7 ± 161.5
**Vit C, mg/d**	167.7 ± 136.8	130.5 ± 83.3	110.1 ± 74.1	186.1 ± 172.1	92.9 ± 68.4 ^d^	110.1 ± 74.1
**Vit E, mg/d**	12 ± 4.6	9 ± 3.7 ^c^	9.1 ± 2.4 ^c^	13.4 ± 6.1	11.2 ± 6	12.8 ± 7
**Alpha tocopherol, mg/d**	6.4 ± 2.6	5.1 ± 2.4 ^d^	5 ± 1.5 ^c^	7.1 ± 3.6	6.1 ± 3.9	7.1 ± 4.2
**Selenium, μg/d**	230.4 ± 96.2	154.3 ± 61.2 ^c^	158.9 ± 66.9 ^c^	245.8 ± 95.9	193.4 ± 66.9 ^c^	187.8 ± 64.9 ^c^

^a^Values are expressed as mean ± SD.

^b^Abbreviations: Sat Fat, saturated fat; MUFA, Monounsaturated fatty acids; PUFA, polyunsaturated fatty acids.

^c^P < 0.001 Comparison with baseline within group.

^d^P < 0.05 Comparison with baseline within group.

## 5. Discussion

Our results indicate that weight reduction might be a good therapeutic strategy for NAFLD in obese patients who achieve weight loss of 9.7% of initial body weight after 6 months of diet therapy.

In our study, 9.7% weight reduction (adherent group) was associated with reduction in BMI, waist circumference, waist to hip ratio (WHR), DBP, total cholesterol, LDL, triglyceride, liver enzymes, and CK 18-M30 levels. Even a mean reduction of only 2% of initial body weight (nonadherent group) during 3 months led to a significant decrease in BMI, waist circumference and waist to hip ratio, although we could not find any significant reduction in whole body inflammation (TNF-α, IL6) and oxidant status (MDA).

Regarding the anthropometric indices, our study results are in line with those of previous ones (16, 19, 22, 27-32); our study is an exclusive nutritional intervention. We observed that 56% of the patients with NAFLD lost more than 5% of their initial weight after 6 months. All anthropometric indices were significantly decreased in those who lost more than 5% of their initial weight, which is in line with other similar studies evaluating the exclusive nutritional intervention in patients with NAFLD. However, the studies assessing lifestyle modifications including both dietary and physical activity recommendations have also shown the similar results.

The results of the present study also demonstrate that weight loss has additional beneficial effect on blood pressure. In particular, the adherent group had a greater reduction in DBP. Shah K et al. reported a significant reduction in systolic blood pressure (SBP) in NAFLD patients with only dietary intervention, and significant reduction in both SBP and DBP in patients with both diet and physical activity intervention (30). Also, Oza et al. (29) showed that lifestyle modification resulted in a significant decrease in both SBP and DBP in patients with NAFLD; however, both of these studies were conducted on older patients. Tomas et al. could not find any significant change in SBP and DBP which might have been due to less weight reduction (4%) in their study population (31). Neither our study, nor other similar studies have evaluated the salt intake of the patients as a covariate in determining the blood pressure changes in these patients. Since there was no recommendation about salt consumption in any group, it seems that weight loss more than 5% of initial weight per se can affect blood pressure in patients with NAFLD.

Among the selected biochemical characteristic total cholesterol, LDL and TG were significantly decreased in adherent group after 6 months of nutritional intervention which is in linewith the result of a similar study (27). There was no significant effect on blood glucose and insulin in the adherent group (16, 27, 31). This data is in line with the study conducted by Thomas et al. (31) who concluded that this was probably due to normal glucose and insulin levels before the intervention; thus, their weight loss did not significantly affect their fasting glucose and insulin levels.

Hung et al. reported that loosing 5% and 7% of total body weight improves liver enzymes and histological changes, respectively (33). In the present study, we found that weight reduction was associated with ALT, AST and GGT improvement within three months (7.1% weight reduction) and 6 months (9.7% weight reduction) follow up in adherent group, but in nonadherent group only 2% weight loss within 3 months was associated with ALT, AST improvement. In our patients, reduction of liver enzymes was correlated with the amount of weight loss without necessarily normal BMI. The reason for this observation is not entirely clear, but may be related to changes in eating habits or dietary component (16). In our study, intake of carbohydrate, total sugar, sucrose, fructose, total fat, saturated fat, and cholesterol was significantly decreased. Therefore, the reduction of liver enzymes might be at least partially due to the reduction in sugar and fat consumption (5, 18, 34). 

To our best knowledge, this is the first study demonstrating that weight loss more than 5% of initial body weight can reduce CK18 significantly. Caspase-cleaved CK18-M30 as a specific measurement of apoptosis is a reliable noninvasive biomarker to monitor disease activity, and to evaluate the therapeutic response of patients with NAFLD (5, 35, 36). It was suggested that CK-18 fragment levels greater than 380.2 U/L can definitely predict NASH (5). It has been demonstrated that SFAs are potentially hepatotoxic through induction of lipoapoptosis (37). So, weight loss can prevent hepatocellular apoptosis in NAFLD through improving fatty acids metabolism (38).

In this study, IL-6 was significantly increased in nonadherent group, and TNF-α tended to slightly decrease in the both groups. Initial reports supported a hepato-protective action of IL- 6 in steatotic liver, but long term IL- 6 exposure may sensitize liver to injury and apoptotic cell death (20, 39, 40). Lang et al. suggested that significantly weight loss and long term weight -control through life style pattern modification for 12 months is necessary for decreasing TNF - α level (27).

Extensive evidence supports a central role of TNF-α and other pro inflammatory cytokines in development of fatty liver, but several reasons might explain the discrepancies.

In fact, in humans, TNF-α has a relatively short half - life and low circulation level, which may not reflect the changes occurring in the liver. Furthermore, we cannot adjust all factors that can influence the level of circulating TNF-α. 

Of course, some factors might have influenced TNF-α level such as difference in the study population. We also did not adjust for other factors that may influence TNF-α circulating level (39). Gene expression of TNF-α and TNF receptor is increased in liver of patients with NASH compared to both normal liver and fatty liver, and expression is higher in those patients with more severe disease (41). A similar trend has been observed for IL-6 level (40).

We measured serum MDA as a lipid peroxidation index with high solubility, and we could not find any significant changes in its serum level in the both groups. MDA was increased in 90% of NASH patients compared to patients with steatosis, illustrating the increase of oxidative stress. MDA, a product of polyunsaturated fatty acids and reactive oxygen species (ROS), is widely used as a marker of lipid oxidant because of its simplicity to assess. Its use in plasma as a biomarker remains controversial since it does not originate exclusively from lipid peroxidation, and is not metabolically stable and the colorimetric determination lacks specificity (42). Diet constituents including antioxidant vitamins can modulate redox reaction and the extent of oxidative stress (43).

Although diet modification in our study resulted in fewer intakes of antioxidant agents such as vitamin E, C and selenium, it could not affect the redox system of the body because all components of dietary intake including antioxidants and oxidants were decreased similarly. On the other hand, no significant correlation has yet been found between any measures of oxidative stress and either grade of inflammation or stage of fibrosis in patients with NAFLD (44).

### 5.1. The Limitations of the Study

Our study had limitations; our patients had both biochemical and ultra sonographic findings indicating NAFLD, but it was not possible to distinguish between simple fatty liver and NASH. Also we did not adjust for other factors that might have influenced TNF-α or IL-6 levels. Low sample size and sampling strategy and inability to generalize findings to target population is another limitation of our study.

The advantage of this study was the measurement of CK18 as a reliable marker of hepatocyte apoptosis, which was measured before and after weight loss. Our results showed that weight reduction can reduce hepatocellular apoptosis and ultimately less hepatic fibrosis.

### 5.2. Summary

In conclusion, our findings support the evidence recommending nutritional intervention for weight loss as the first step in the management of patients with NAFLD.
